# Molecular Imaging in Stem Cell Therapy for Spinal Cord Injury

**DOI:** 10.1155/2014/759514

**Published:** 2014-02-19

**Authors:** Fahuan Song, Mei Tian, Hong Zhang

**Affiliations:** ^1^Department of Nuclear Medicine, The Second Affiliated Hospital of Zhejiang University School of Medicine, 88 Jiefang Road, Hangzhou, Zhejiang 310009, China; ^2^Zhejiang University Medical PET Center, Zhejiang University, 88 Jiefang Road, Hangzhou, Zhejiang 310009, China; ^3^Institute of Nuclear Medicine and Molecular Imaging, Zhejiang University, 88 Jiefang Road, Hangzhou, Zhejiang 310009, China; ^4^Key Laboratory of Medical Molecular Imaging of Zhejiang Province, 88 Jiefang Road, Hangzhou, Zhejiang 310009, China

## Abstract

Spinal cord injury (SCI) is a serious disease of the center nervous system (CNS). It is a devastating injury with sudden loss of motor, sensory, and autonomic function distal to the level of trauma and produces great personal and societal costs. Currently, there are no remarkable effective therapies for the treatment of SCI. Compared to traditional treatment methods, stem cell transplantation therapy holds potential for repair and functional plasticity after SCI. However, the mechanism of stem cell therapy for SCI remains largely unknown and obscure partly due to the lack of efficient stem cell trafficking methods. Molecular imaging technology including positron emission tomography (PET), magnetic resonance imaging (MRI), optical imaging (i.e., bioluminescence imaging (BLI)) gives the hope to complete the knowledge concerning basic stem cell biology survival, migration, differentiation, and integration in real time when transplanted into damaged spinal cord. In this paper, we mainly review the molecular imaging technology in stem cell therapy for SCI.

## 1. Introduction 

Spinal cord injury (SCI), which results from trauma or progressive neurodegeneration, is a devastating and life-altering injury. It often affects young and healthy individuals who are suffering from severe functional and sensory deficits. This debilitating condition not only creates enormous physical and emotional cost to individuals but also is a significant financial burden to the society. The annual incidence of SCI is 15–40 cases per million worldwidely [1].

SCI is mainly divided into two types: traumatic SCI and nontraumatic SCI. A global-incident rate (2007) of traumatic SCI is estimated at 23 traumatic SCI cases per million [2]. The most common causes of traumatic SCI are road traffic accidents, falls, occupational mishaps, and sports-related injuries [3]. Most SCI occurs at the cervical level (approximately 55%) with a mortality of 10% in the first year following injury and an expected lifespan of only 10 to 15 years after injury. Thoracic, thoracolumbar, and lumbosacral level injury each accounts for approximately 15% of SCI [3].

Despite the progress of medical and surgical management as well as rehabilitation approaches, many SCI patients still experience substantial neurological disabilities [4, 5]. Moreover, clinical trials of pharmacologic therapeutics within the last two decades have either failed to prove efficacy or provided only modest reductions in functional deficits [6–8].

Previous researches on SCI mainly focused on improving neurological manifestations of SCI while ignoring the pathological changes of spinal cord. According to the progress, SCI could be divided into primary injury phase which is the physical injury and secondary injury phase [9, 10]. The primary injury phase damages both upper and lower motor neurons and disrupts sensory, motor, and autonomic (including respiration, cardiac output, and vascular tone) functions. The secondary injury phase is the amplification of the original injury with a subsequent cascade of molecular and cellular events [11]. Pathophysiological processes occur after the primary injury phase and are rapidly instigated in response to the primary injury in order to control and minimize the damage. However, these are largely responsible for exacerbating the initial damage and creating an inhibitory microenvironment which prevent endogenous efforts of regeneration and remyelination. Such secondary processes include ischemia, inflammation, lipid peroxidation, disruption of ion channels, fluid and electrolyte disturbances, producing of free radicals, axonal demyelination, necrosis, glial scar formation, and apoptosis [12, 13]. Nevertheless, endogenous repair and regeneration happen during the secondary phase of SCI to minimize the extent of the lesion, reorganize blood supply through angiogenesis, clear cellular debris, reunite and remodel damaged neural circuits, and offer exploitable targets for therapeutic intervention [14, 15]. Thus, these secondary damages are crucial to SCI therapy.

Increasing interest has focused on the development of innovative therapeutic methods that aim to regenerate damaged CNS tissue by taking advantages of recent advances in stem cell and neuroscience research [25, 26]. Preclinical models demonstrated that stem cell transplantation could ameliorate some secondary events of SCI through neuroprotection and restore lost tissue through regeneration [27]. Cumulative researches have demonstrated the feasibility of stem cell therapy and various stem cells have been used to protect against the secondary damage with enhancing the regeneration of a damaged spinal cord. Thus, stem cell transplantation would be one of the promising approaches for the regeneration of an injured spinal cord [28].

Recent studies suggested that stem cell therapy could improve neural function in SCI by replacing damaged cells [29, 30]. Therefore, it becomes more and more important to explore the detailed mechanisms of stem cell therapy for SCI and monitor the fate of these cells *in vivo*, including survival, migration, distribution, rejection, integration, and differentiation.

Fortunately, molecular imaging gives an effective way for such research. Well-established imaging modalities used by researchers for the purpose of molecular imaging are positron emission tomography (PET), magnetic resonance imaging (MRI), whereas others have employed newer modalities based on the transmission of light through tissues, such as *in vivo* bioluminescence imaging (BLI) and fluorescence imaging (FLI). The label of stem cells requires labelling methods making grafted cells distinguishable from host cells to follow transplanted stem cells by the above methods.

PET is a nuclear imaging technique that produces a three-dimensional image or picture of functional processes in the body. The system detects pairs of gamma rays emitted indirectly by a positron-emitting radionuclide (tracer), which is introduced into the body on a biologically active molecule. It can provide metabolic changes and be used for tracing radioactively labeled stem cells *in vivo*. MRI can be used for noninvasive tracking of transplanted cells which are usually labeled with super-paramagnetic nanoparticles as contrast agents in longitudinal studies on living animals. Gadolinium and ferric oxide are two common contrast media used for cell labeling in MRI [31]. Recently, there were several studies using MRI to trace the transplanted stem cells in SCI models [32–34]. Their results showed that the transplanted stem cells could be readily detected *in vivo* using noninvasive MRI techniques. BLI is a newly developing technology for dynamically observing biological behavior. It is based on an enzymatic light production system. The enzyme in action is a luciferase which uses the substrate luciferin to produce light. Then the emitted light is subsequently captured by a highly sensitive CCD camera. Using this technology, cells should be transgenically modified to steadily express luciferase. Then, cells can be tracked in a long periods of time because luciferase expression is preserved during proliferation so that all cells and their descendants express luciferase without dilution. Thus, we can observe the stem cells' dynamic behavior *in vivo* through this method.

## 2. Stem/Progenitor Cell Therapy in SCI

### 2.1. Mechanism of Stem Cell Therapy

According to the pathophysiological targets of SCI, transplanted cells should satisfy the following requirements. Firstly, enable regenerating axons to cross any cysts or cavities; secondly, functionally replace dead cells; thirdly, create an environment to support axonal regeneration and myelination. Stem or progenitor cells are capable of modifying the lesion environment, providing structural support, myelination, increasing neurotrophic factors for neuroprotection, and endogenous activation [35–38]. As a result, stem cells have potential for remyelinating lesions and are an attractive cell source for cell therapy of SCI. And recent experimental studies suggested that stem cell therapy can improve neural function in SCI (Table 1). The opportunity to enhance endogenous adaptability through cell-based approaches has led to a great interest in developing stem cell transplantation therapies that could potentiate and synergise with other treatment modalities to maximise neuroplasticity and produce meaningful recovery.

Characteristics and purported mechanisms of action of stem cells will then be discussed, with specific attention paid to axonal regeneration and regrowth, growth factor release, guidance through inhibitory cues, remyelination, and induction of anatomical neuroplasticity.

### 2.2. Bone Marrow Stem Cells

Transplantation of bone marrow-derived mesenchymal stem cells (BMSCs) for SCI has been previously reviewed [39–43]. Many studies have examined BMSCs in SCI rodents and the results showed improved locomotor recovery [44–46].

There are two types of adult bone marrow stem cells: hematopoietic stem cells (HSCs) and mesenchymal stem cells (MSCs). Zhu et al. compared various properties of human umbilical cord-derived MSCs (HUCMSCs) with human placenta-derived MSCs (HPDMSCs), including cell proliferation, apoptosis, cellular morphology, ultrastructure, and their ability to secrete various growth factors. Their findings indicate that different sources of MSCs have different properties and that care should be taken when choosing the appropriate sources of MSCs for stem cell transplantation [47]. In rodent studies, BMSCs was able to promote a certain degree of axonal regrowth and sprouting, at least in transection models [48]. Lee et al. have used human umbilical cord blood derived mesenchymal stem cells (hUCB-MSCs) to treat dogs with SCI and observed its long-term effects on dogs. It was found that hind-limb recovery in 4 dogs among the five transplanted dogs was significantly improved and the results suggest that transplantation of hUCB-MSCs may have beneficial therapeutic effects [49].

The grafting of MSCs to treat SCI has shown promising results in animals; however, less is known about the effects of autologous MSCs in human SCI. Park et al. explored 10 SCI patients who underwent intramedullary direct MSCs transplantation into injured spinal cords. All patients did not experience any permanent complication associated with MSC transplantation. Three patients showed gradual improvement in activities of daily living, changes on magnetic resonance imaging such as decreases in cavity size and the appearance of fiber-like low signal intensity streaks, and electrophysiological improvement [50].

Despite these potential benefits, there are reported adverse effects of MSCs, such as increased recurrence of hematological malignancies and enhanced tumor growth and metastases [41, 51, 52]. And more preclinical trials of MSC-based therapy need to be preformed.

### 2.3. Neural Stem Cells and Neural Progenitor Cells

Neural stem cells (NSCs) have been classified as a kind of neural lineage stem cell which is able to self-renew and to give rise to all types of mature neural cells including neurons, astrocytes, and oligodendrocytes [53, 54]. The isolation of adult neural stem cells in mammals was first reported in 1992 by Reynolds and Weiss [55]. NSCs for therapeutic applications are derived from ESCs and progenitor cells are isolated from fetal tissue. Neural progenitor cells (NPCs), like stem cells, have a tendency to differentiate into a specific type of cells.

NSCs and NPCs are found in both fetal and adult CNS [56]. Adult NPCs can be typically harvested from the subventricular zone of the brain or the spinal cord. Embryonic NPCs can be taken from the CNS of rodent embryos and expanded as neurospheres. They all contain precursors for neurons, astroglia, and oligodendrocytes plus stem cells capable of self-renewal [57]. Salazar and his colleagues injected human neural stem cells (hCNS-SCns) into immunodeficient NOD-scid mice 30 days after spinal cord contusion injury in order to test the ability of hCNS-SCns to survive, differentiate, migrate, and promote improved locomotor recovery [54]. They found that hCNS-SCns can survive, differentiate, and promote locomotor recovery when transplanted into an early chronic injury microenvironment. These results suggest that hCNS-SCns graft has efficacy in an early chronic SCI location and expands the “window of opportunity” for intervention.

The extent of glial scar formation and the characteristics of inflammation are the most remarkable difference in the injured spinal cord microenvironment between the subacute and chronic phases. By contrast, the distribution of chronically grafted NSPCs was restricted compared to NSPCs grafted at the subacute phase because a more prominent glial scar located around the lesion epicenter enclosed the grafted cells. Therefore, glial scar formation and inflammatory phenotype should be considered in order to achieve functional recovery by NSPCs transplantation in cases at the chronic phase [58]. Another study showed that grafted NSPCs mainly differentiate into astrocytes after transplantation at the acute phase [59]. Inflammatory cytokines, such as IL-6, TNF-*α*, and CXCL1, also increase remarkably in the injured spinal cord [60]. These cytokines might induce NSPCs to differentiate into astrocytes. Furthermore, growth factors like EGF and NT3 have demonstrated beneficial effects on the survival of NSPCs [61, 62].

When transplanted into mice in an animal model of SCI, human NSCs promoted locomotor recovery [63]. The study of Mothe indicated that multipotent NSPCs can be delivered from the adult human spinal cord of organ transplant donors and that these cells differentiate into both neurons and glia following transplantation into rats with SCI [64]. In addition, human fetal brain modified NSPCs transplanted subacutely into the contused cervical spinal cord of adult common marmosets produced significantly greater grip strength than controls [65]. Researchers have shown that these NSCs and NPCs primarily differentiate into oligodendrocytes *in vitro* [66] and *in vivo* [67, 68]. Recently, researchers demonstrated that self-renewing multipotent NSCs and NPCs could be acquired from the adult human spinal cord of organ transplant donors and that these cells differentiate into both neurons and glia following transplantation into rats with SCI [64].

Simultaneously, the aims of axonal regeneration through the injury area have been replaced preclinically by more realistic objectives of remyelination and provision of trophic support for endogenous precursors and axons. It makes NPCs much more promising candidates of cell therapy for SCI and probably heralds their increased use in clinical trials.

### 2.4. Embryonic Stem Cells

Embryonic stem cells (ESCs) are pluripotent cells derived from the inner cell mass of developing blastocyst embryos that can differentiate into nearly all cell types [69]. Notably, ESCs have great developmental plasticity and can be induced to become NSPCs with specific differentiation potentials, making them a major candidate of cell replacement therapies for SCI. Mcdonald et al. transplanted neural differentiated mouse embryonic stem cells into rats' spinal cord after traumatic injury. The cells that transplanted into the injured spinal cord differentiated into neurons, astrocytes, and oligodendrocytes and showed partial functional recovery [70]. As noted above, SCI causes extensive demyelination and oligodendrocytes are particularly vulnerable to apoptosis. ESCs predifferentiated into oligodendrocyte progenitor cells (OPCs) remyelinated spared axons and improved recovery when transplanted subacutely into the injured rat spinal cord [71, 72]. The studies document the feasibility of predifferentiating hESCs into functional OPCs and demonstrate their therapeutic potential after SCI.

### 2.5. Induced Pluripotent Stem Cells

The discovery of induced pluripotent stem cells (iPSCs) has opened a new potential therapeutic approach for regenerative neuroscience, although iPSCs have not yet been used clinically in SCI cell therapy. iPSCs were developed in 2006 by Takahashi and Yamanaka, who showed that mouse somatic cells, such as fibroblasts, could be reprogrammed to pluripotency with retroviral expression of four transcription factors OCT4, SOX2, KLF4, and c-MYC and achieve similar morphology, pluripotency, self-renewal, and gene expression as ESCs, without the requirement for an embryo [73].

The clinical use of ESCs is complicated; however, by ethical and immunological concerns, both of which might be overcome by using pluripotent stem cells which can be derived directly from a patient's own somatic cells [74]. Experimental studies using iPSCs-derived neurospheres transplanted subacutely after contusion SCI showed remyelination, axonal outgrowth of serotonergic fibers, and promotion of locomotor recovery.

Different kinds of iPSCs have different performance in SCI treating. Transplantation of “unsafe” iPS-derived neurospheres may result in teratoma formation and sudden loss of locomotor function [21]. Kramer's review highlights emerging evidence that suggests that iPSCs are not necessarily indistinguishable from ESCs and may occupy a different “state” of pluripotency with differences in gene expression, methylation patterns, and genomic aberrations which may reflect incomplete reprogramming and may therefore impact on the regenerative potential of these donor cells in therapies [75]. Furthermore, the first study on iPSC-derived chimeric mice demonstrated that they were prone to cancer and attributed this property to the reexpression of the c-myc reprogramming factor [76]. In brief, iPSCs are likely to carry a higher risk of tumorigenicity than ESCs, due to the inappropriate reprogramming of these somatic cells, the activation of exogenous transcription factors [77, 78]. Thus, safe iPSC-derived clones would need to be screened and selected [21, 78].

Undifferentiated IPSCs, like ESCs, have high tumourigenicity in pathological conditions [79]. Therefore, the safety of IPSCs should be evaluated before clinical IPSCs-based therapy. Of note, Tsuji et al. recently derived “safe” mouse iPSCs and observed trilineage neural differentiation and functional recovery in a contusion model of SCI without teratoma formation [21].

## 3. Molecular Imaging in Stem Cell Therapy for Spinal Cord Injury

Transplantation of stem cells has a good prospect of clinical application. However, the challenges in the field of molecular imaging are to develop effective imaging strategies with reporter systems and probes which should firstly reveal cellular and molecular processes throughout an entire study period. Secondly, probes should be highly sensitive to small changes in cell function and distribution. Finally, they do not alter the labeled biological process itself significantly. Molecular imaging technologies will greatly facilitate the functional monitoring and evaluation of a wide range from genes to organs for their roles in health and disease. Herein, molecular imaging has served as a platform to test stem cell therapy for SCI.

### 3.1. Stem Cell Labelling

Cell labeling can be divided into two types: physical cell labeling and reporter gene imaging. Physical cell labeling is completed before cell administration and can be accomplished with superparamagnetic iron oxide (SPIO) particles for MRI [83, 84], radionuclide labeling for SPECT [85], and PET, nanoparticle labeling for fluorescent imaging [86]. In reporter gene imaging, a gene coding for the synthesis of a detectable protein is introduced into a target cell line or tissue via viral or nonviral vectors. Because the reporter gene integrates into the host cell's chromosome following stable transfection or transduction, the reporter gene is expressed by progeny. As a result, changes in signals following cell administration can be used as indicators of cell proliferation and cell death [87].

### 3.2. Positron Emission Tomography

Positron emission tomography (PET) imaging, especially ^18^FDG PET imaging, has been used mainly in cancer [88–90]. PET uses positron emitting radioisotopes as probes for imaging cells *in vivo* to monitor labeled stem cells and it also has been applied widely to detect and quantify subtle abnormalities in CNS diseases. PET has served as a platform to test stem cell therapy for neurological disease allowing more rapid progression in both preclinical and clinical studies. In order to explore the effect of *in vivo* PET in tracking the stem cells transplanted into spinal cord, Bai et al. transplanted human neural progenitor cells into rabbits' injured spinal cord; rabbits were injected with ^11^C-raclopride intravenously and then underwent PET imaging [91]. ^11^C-raclopride PET imaging of the live rabbits showed accumulation of radioactivity at the hNPC-TERT cell injection site with a standard uptake value significantly higher than that of control group (the HeLa cell transplantation group) (*P* < 0.01). ^11^C-raclopride PET imaging of the isolated spinal cords showed rounded focal image of increased radioactivity in the hNPC-TERT cell transplantation group and linear image of radioactivity without clear border in the HeLa cell transplantation group. Meanwhile, fluorescent microscopy showed the same results. These results suggested that PET with radioisotope labeled tracer is useful for functional studies in developing cell-based therapies.

### 3.3. Magnetic Resonance Imaging

**Figure 1:**
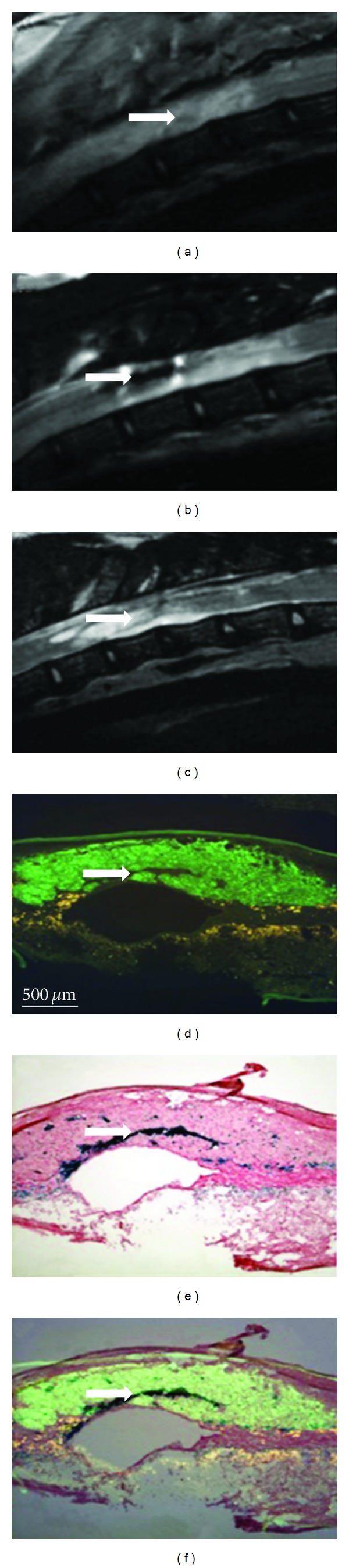
The T2-weighted MR images of the injured rat's spinal cord before and after SPC-01 cell transplantation. The white arrows show labeled transplanted cells and lesion site. (a) The T2-weighted MR images of a spinal cord lesion 5 days after lesion induction before transplantation. (b) Spinal cord with a cell graft 8 weeks after cell transplantation. (c) Control spinal cord lesion 8 weeks after saline injection. (d) Two serial sections were stained with the human mitochondrial marker (MTCO2). (e) The same sections of D were stained with iron. (f) Overlay of MTC02 and iron staining [80].

Magnetic resonance imaging (MRI) is a widely used and powerful imaging technique which can provide high resolution and 3D anatomical imaging. It can be used to evaluate the reparation of the injured spinal cord [92]. For monitoring the efficiency of cell transplantation, cellular homing, or targeting, grafted cells can be labeled with superparamagnetic iron oxide nanoparticles (SPION) and detected by means of MRI. In a study of NPC transplantation, SPIO labeling does not affect NPCs' survival and differentiation potential *in vitro* and labeled NPCs were found migrating along white matter tracts as demonstrated by MRI [93]. In addition, cells labeled with SPION can be manipulated in a magnetic field. In research of Vanecek et al., intrathecally transplanted cells labeled with SPION were guided by a magnetic field and successfully targeted near the lesion site in the rat spinal cord. The results showed that targeting efficiency could be increased by using magnets that produce spatially modulated stray fields [84]. Such magnetic systems with tunable geometric parameters may provide the additional level of control needed to enhance the efficiency of stem cell delivery in injured spinal cord. Hu et al. used labeled human umbilical cord mesenchymal stem cells (hUC-MSCs) *in vivo* for tracking hUC-MSCs' fate with noninvasive MRI [94]. SPIO was added to cultures at concentrations equivalent to 0, 7, 14, 28, and 56 mg Fe/ml and incubated for 16 h. *In vivo* MRI 1 and 3 weeks after injection showed a large reduction in signal intensity in the region transplanted with SPIO-labeled hUC-MSCs. The results showed that noninvasive imaging of transplanted SPIO-labeled hUC-MSCs is feasible. Recently, Amemori et al. used neural stem cell line derived from human fetal spinal cord tissue (SPC-01) to treat a balloon-induced SCI [80]. SPC-01 cells labeled with poly-L-lysine-coated SPION were implanted into the lesion 1 week after SCI. Then T2-weighted images were obtained (Figure 1). The transplanted animals displayed significant motor and sensory improvement 2 months after SCI, when the cells robustly survived in the lesion and partially filled the lesion cavity.

### 3.4. Optical Imaging

**Figure 2:**
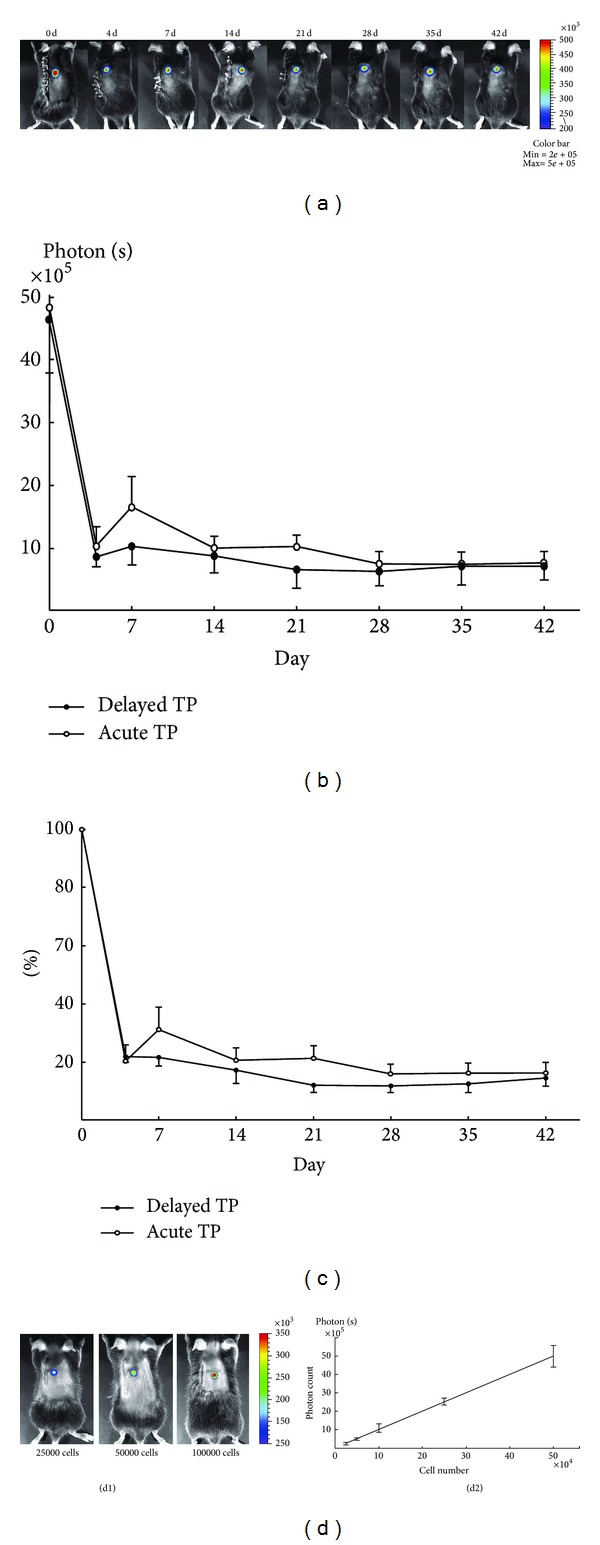
The time course of viability of transplanted NSPCs for SCI. (a) Images of a representative mouse that received acute transplantation (TP) of luciferase-expressing NSPCs confirmed long-term cell viability. Drastic reductions in signal intensity within the first 4 days after transplantation and then relatively stable bioluminescent signals for the following 6 wk were observed in both acute and delayed transplantation groups. There were no differences between acute and delayed transplantation groups in both value of signal intensity (b) and the rate to initial value (c) at each time point. Values are means ± SE (*n* = 8). (d) The correlation between grafted cell numbers and luminescent intensity was confirmed *in vivo*. Values are means ± SE (*n* = 4) [59].

Bioluminescence imaging (BLI) uses luciferase-transduced transplanted cells that can be detected noninvasively *in vivo* by virtue of their reporter gene, which is expressed only when cells are alive [95, 96]. Luciferase (Luc) gene is the most commonly used gene for *in vivo* luminescence which is obtained from the North American firefly. This Luc gene encodes a 550 amino acid protein, Luc. Considering the near absence of endogenous light from mammalian cells and tissues, luciferases have a significant advantage as optical indicators in live mammalian cells and tissues, the inherently low background [97]. It is a powerful tool for the detection of exclusively living grafted cells that stably express luciferase in living animals after administration of luciferin (the substrate of luciferase) in the presence of oxygen and ATP as a source of energy [98]. Using the luciferase reaction *in vivo* as a marker of gene expression requires that the substrate is nontoxic and well distributed to the animals' tissues after exogenously adding (usually via intraperitoneal injection). At present, no humoral immune responses, hepatic toxicity, or germ-line integration were observed in models of luciferase reaction. Studies have also suggested that luciferin could be distributed throughout the entire animal and even could not be restricted by the blood-brain or placental barriers [98]. Previous studies demonstrated that the number of photons which emitted from the labeled cells and transmitted through murine tissues was sufficient to detect 1–2.5 × 10^3^ cells in the peritoneal cavity, 1 × 10^4^ cells at subcutaneous sites, and 1 × 10^6^ circulating cells immediately following injection [99, 100]. Recently, noninvasive bioluminescent imaging was successfully applied to investigate the survival of neural stem/progenitor cells following transplantation into the lesioned mouse spinal cord. It was found that the intralesional application of NSPCs among 3 different procedures (intralesional, intrathecal, and intravenous injection) is the most effective and feasible method for transplanting NSPCs into the SCI site [101]. Okada et al.'s study is a well example of stem cell transplantation study [59]. The third-generation lentiviral vectors enabled efficient transduction and stable expression of both luciferase and a variant of green fluorescent protein in primary cultured NSPCs. Signals from these cells were detectable for up to 10 months or more after transplantation into the injured spinal cords of C57BL/6J mice. Analysis of both acute and delayed transplantation groups revealed drastic reductions in signal intensity within the first 4 days after transplantation, which was followed by a relatively stable bioluminescent signal for 6 wk (Figure 2).

### 3.5. Multimodality Imaging

**Figure 3:**
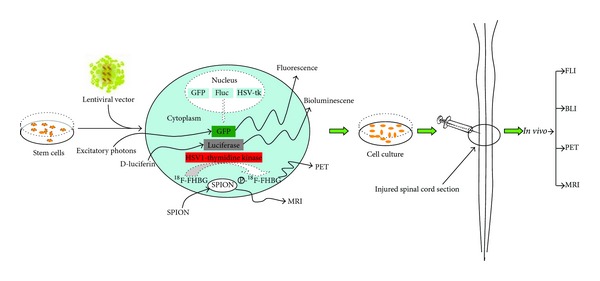
Multimodality of imaging can be applied for tracking stem cell behavior. A work flow chart for labeling cells and introducing labeled cells *in vivo*: firstly, cells are labeled using a marker for positron emission tomography (PET), magnetic resonance imaging (MRI), bioluminescence imaging (BLI), or fluorescence imaging (FLI). Secondly, cells are cultured *in vitro* and injected into the injured spinal cord. Finally, stem cells are then tracked *in vivo* with a camera or scanner.

No single imaging modality can provide all the information required to track transplanted stem cells and monitor their functional effects. Every imaging modality for stem cell tracing has its own advantages and disadvantages [81, 82] (Table 2). PET has a high sensitivity in tracking biomarkers *in vivo* but lacks the ability of providing detailed anatomic structures. SPIO-labeled stem cells can be detected by means of MRI and get high resolution and 3D anatomical imaging; however, the low sensitivity limits its application on cell tracing. Furthermore, cell division may dilute intracellular markers and then the signals may fail to reflect cells' number and location due to shedding of iron particles [102]. Hence, it is necessary to combine complementary imaging methodologies. Multimodality imaging approaches may minimize the potential drawbacks of using each imaging modality alone and a tailored combination of 2 or more techniques may be the best approach for a given experiment (Figure 3).

Computed tomography (CT) scanners can overcome the lacks of anatomic structures. PET and CT scanners can be used in conjunction to get more detailed, fused images. In a research of Nandoe Tewarie RD, PET was used in combination with CT imaging techniques for longitudinal monitoring of the injured spinal cord. With PET-CT, combined with a simulation-based partial-volume compensation (PVC) method, they can serially measure standardized uptake values of the T9 and T6 spinal cord segments and reveal small, but significant, differences [103]. Because MRI has limitations in determining the viability of labeled transplanted cells, another imaging modality is required. The combination of MRI and PET also allows the acquisition of anatomical, physiological, and metabolic information, all from the same subject [104, 105]. By utilizing CT information acquired by an X-ray detector, the 3-dimensional location can be reconstructed using some bioluminescent tomography (BLT) reconstruction methods such as the adaptive finite element method and Bayesian method [82]. Researchers combined MRI with BLI to simultaneously monitor the location and viability of transplanted cells *in vivo* within the same animal. In recent study, Kim et al. evaluated the therapeutic effects of transplanted human glial precursor cells (hGPs) together with the *in vivo* fate of these cells using MRI and BLI [106]. In order to determine their potential for therapy of multiple sclerosis (MS), they used MRI and BLI side-by-side as complementary imaging techniques to evaluate the effects of transplanted hGPs on experimental autoimmune encephalomyelitis (EAE) pathogenesis. The results demonstrated that intracerebroventricularly (ICV) transplantation of short-lived hGPs can have a remote therapeutic effect through immunomodulation from within the ventricle, without cells directly participating in remyelination.

There are also some problems of nonspecific signals obtained with the different imaging modalities as a result of grafted cells that have died. For example, grafted cells prelabeled with iron oxide nanoparticles that have died postgrafting may result in MRI signals representing macrophage phagocytosis of labeled cell debris. However, PET can use report gene system to avoid such disadvantage, and the high detection sensitivity of PET imaging techniques could offset nonspecific signals of MRI. Hybrid PET/MR imaging might present a formidable technical and suggest potential spinal cord applications exploiting unique properties of the combined instrumentation.

## 4. Perspective and Summary

The potential of stem cells to reconnect the injured spinal cord and repopulate the area of injury has fascinated SCI researchers. Although some authors believe that endogenous remyelination is effective albeit somewhat slower. The field has learned a great deal about evaluating the fate of stem cells once they are implanted into the cord. How many survive? Do they integrate and migrate? How do they influence the host microenvironment? More and more evidence suggest that the stem cells themselves may be the source of growth factors and have remarkable influence on the injured microenvironment.

The interest in stem cell transplantation for SCI will remain high. This field will obviously continue to evolve, with hopes that further refinement, and understanding will increase the chances that cell transplantation will someday emerge as a fruitful treatment for patients. The complexities of attenuating the tissue damage and secondary complications due to trauma and reconstructing the cytoarchitecture of the injured spinal cord are very challenging. Hopefully, the rapid advances being made in stem cell biology will result in effective experimental and clinical trials of stem cell therapy for SCI.

Molecular imaging is a new discipline which makes it possible *in vivo* of cellular and molecular aspects of pathophysiological processes and therapeutic interventions. PET has high flexibility for the production of specific probes for the detection of different processes in the living subject. However, the production of PET needs advanced chemistry and tight quality control. MRI is also a contending and complementing modality in molecular imaging essential for stem cell studies. It can be used to evaluate the reparation of the injured spinal cord as well as tracing labeled stem cell *in vivo*. Optical imaging has a high molecularly sensitivity, but it provides lesser anatomical localization and is mainly used in small animals.

In summary, the usage of stem cell treatment might restore axonal continuity, connect the area for axonal regeneration, and promote axonal growth back to its distal targets. And the use of a noninvasive imaging method would have the advantage that stem cells transplanting in individual animals can be monitored longitudinally. The multimodality imaging technique allows for studying acute pathological events following a spinal cord lesion, and the development of implanted spinal chamber enables long-term imaging for chronic spinal cord preparations. Therefore, *in vivo* imaging allows the direct observation of dynamic regenerative events of individual stem cells after traumatic injury in the living subject [107]. We predict that future improvement in molecular imaging will make important contributions to our understanding of stem cells' transplantation and allow us to assess the therapeutic effect on a molecular scale.

## Figures and Tables

**Table 1:** Stem cell-based cell therapy in experimental SCI models.

Cell type	Cell number	Route	Time after SCI	Weeks after cell injection	Host animal	Cotreatment method	Functional outcome	References
BMSCs	5 × 10^6^	IL	24 h	3	Rabbit	BMSCs were Ngb gene-modified	Significant functional improvement	[16]
OPCs	5 × 10^5^	IL	3 d	4	Rat	No	Functional improvements in SSEP amplitudes and latencies	[17]
iPSC-NS/PCs	1 × 10^6^	IL	9 d	12	Marmoset	No	Promoted functional recovery	[18]
NSCs + OECs	3 × 10^5^	IL	Immediately	4	Rat	Cotransplantation of NSCs and OECs	Improve sensory function	[19]
ESCs	5 × 10^5^	IV	2 h	4	Mice	No	Promoted hind-limb recovery	[20]
iPSCs	5 × 10^5^	IL	9 d	6	Mice	No	Promoting locomotor function recovery	[21]
NS/PCs	8 × 10^4^	IL	9 d	8	Rat	Coinjected with HAMC	Enhanced tissue benefit and functional recovery	[22]
EMSCs	5 × 10^4^	IL	0.5 h	12	Rat	Coinjected with fibrin scaffolds	Improve the behavioral and histological recovery	[23]
BMSCs	1 × 10^7^	IL	7 d	4	Dog	No	Improved functional recovery	[24]

IL: intralesional injection, IT: intratheca injection, IV: intravenous injection, BMSCs: bone marrow mesenchymal stem cells, Ngb: neuroglobin, SSEPs: somatosensory evoked potential, iPSC-NS/PCs: induced pluripotent stem cell-derived neural stem/progenitor cells, HUCBCs: human umbilical cord blood cells, hES: transplanted human embryonic stem, OPCs: cell-derived oligodendrocyte progenitor cells, ESCs: embryonic stem cells, iPSCs: induced pluripotent stem cells, NSPCs: neural stem/progenitor cells, HAMC: hydrogel blend of hyaluronan and methyl cellulose, and EMSCs: ectomesenchymal stem cells.

**Table 2:** Advantages and limitations of different imaging methods for detection of grafted stem cells (modified from Modo et al. [81] and Spiriev et al. [82]).

Imaging modality	PET	MRI	BLI	FLI
Depth of penetration	No limit	No limit	1-2 mm	<1 mm
Spatial resolution	1-2 mm	10–100 *μ*m	Several mm	2-3 mm
Temporal resolution	sec–min	min–hrs	sec–min	sec–min
Imaging agents	Radionuclide labeled compound	Gadolinium, dysprosium iron oxide particles	Luciferins	Fluorescent protein
Toxicity	No	Yes	No	No
Time range of detection	6–12 months	1-2 months	2–8 weeks	Long-term
Detection limits in terms of cell numbers in vivo	1 × 10^4^–1 × 10^5^	5 × 10^5^–1 × 10^6^	1 × 10^3^–1 × 10^6^	2 × 10^4^–5 × 10^5^

Sec: second, min: minute, and hrs: hours.

## Abbreviations

SCI: Spinal cord injury
CNS: Center nervous system
PET: Positron emission tomography
MRI: Magnetic resonance imaging
BLI: Bioluminescence imaging
FLI: Fluorescence imaging
CCD: Charge-coupled device
BMSCs: Bone marrow-derived mesenchymal stem cells
HSCs: Hematopoietic stem cells
MSCs: Mesenchymal stem cells
HUCMSCs: Human umbilical cord-derived MSCs
HPDMSCs: Human placenta-derived MSCs
hUCB-MSCs: Human umbilical cord blood derived mesenchymal stem cells
NSCs: Neural stem cells
NPCs: Neural progenitor cells
hCNS-SCns: Human neural stem cells
NSPCs: Neural stem or progenitor cells
NOD-scid: Nonobese diabetic-severe combined immunodeficiency disease
IL-6: Interleukin-6
TNF-*α*: Tumor necrosis factor-alpha
CXCL1: Chemokine (C-X-C motif) ligand 1
EGF: Epidermal growth factor
NT3: Neurotrophin-3
ESCs: Embryonic stem cells
OPCs: Oligodendrocyte progenitor cells
iPSCs: Induced pluripotent stem cells
OCT4: Octamer-binding transcription factor-4
SOX2: SRY-related high-mobility-group (HMG)-box protein-2
Klf4: Kruppel-like factor-4
c-MYC: Cancer-MYC
SPIO: Superparamagnetic iron oxide
SPECT: Single photon emission tomography
hNPC-TERT: Telomerase-immortalized human neural progenitor cells
SPION: Superparamagnetic iron oxide nanoparticles
hUC-MSCs: Human umbilical cord mesenchymal stem cells
SPC-01: Neural stem cell line derived from human fetal spinal cord tissue
CT: Computed tomography
PVC: Partial-volume compensation
BLT: Bioluminescent tomography
hGPs: Human glial precursor cells
MS: Multiple sclerosis
EAE: Experimental autoimmune encephalomyelitis
ICV: Intracerebroventricularly
IL: Intralesional injection
IT: Intratheca injection
IV: Intravenous injection
Ngb: Neuroglobin
SSEPs: Somatosensory evoked potential
iPSC-NS/PCs: Induced pluripotent stem cell-derived neural stem/progenitor cells
HUCBCs: Human umbilical cord blood cells
hES: Transplanted human embryonic stem
OPCs: Cell-derived oligodendrocyte progenitor cells
HAMC: Hydrogel blend of hyaluronan and methyl cellulose
EMSCs: Ectomesenchymal stem cells
sec: Second
min: Minute
hrs: Hours.
